# A Case of Anomalous Right Coronary Artery With Catastrophic Presentation

**DOI:** 10.7759/cureus.63705

**Published:** 2024-07-02

**Authors:** Farhana Anwar, Ethan Hiles, Maham Shahid, Suzanne Martinez, Mubbasher Syed

**Affiliations:** 1 Internal Medicine, Health Corporation of America (HCA) Florida Orange Park Hospital, Orange Park, USA; 2 Endocrinology, Health Corporation of America (HCA) Florida Orange Park Hospital, Orange Park, USA; 3 Cardiology, Health Corporation of America (HCA) Florida Orange Park Hospital, Orange Park, USA

**Keywords:** anomalous coronary artery with inter-arterial course, anomalous coronary artery, inter-arterial course, anomalous right coronary artery, left coronary cusp, right coronary artery (rca)

## Abstract

Anomalous coronary artery is a rare but potentially life-threatening alteration in the coronary vascular system that is related to an increased risk of myocardial ischemia, ventricular arrhythmias, heart failure, and sudden cardiac death (SCD).

Here, we present the case of a young male who presented to the hospital after a witnessed sudden cardiac arrest. Bystander cardiopulmonary resuscitation was started immediately, and normal sinus rhythm was achieved after electrical cardioversion three times. He was admitted to the ICU for further care upon admission. A CT of the chest showed a potential vascular structure in between the aorta and the pulmonary trunk. He underwent cardiac catheterization, which identified minimal coronary artery disease with the anomalous takeoff of the right coronary artery from the left coronary cusp. A cardiac CT scan obtained also showed an anomalous right coronary artery (ARCA) with an inter-arterial course. After explaining available treatment options and obtaining informed consent, a surgical correction by cardiothoracic surgery was performed using the coronary artery bypass graft technique. The patient recovered well after the surgery and was discharged home. After two years of follow-up, he continued to live life normally without any symptoms.

Early and accurate diagnosis of an anomalous coronary artery is imperative for timely intervention, as malignant coronary artery diseases can often have a catastrophic presentation with acute coronary syndromes, myocardial infarction, or SCD. We present here a case of successful diagnosis of ARCA and its prompt surgical correction using coronary artery bypass grafting technique in a young adult. Despite the availability of various other treatment options, our case underscores coronary artery bypass grafting as a viable choice for individuals with anomalous coronary arteries, particularly in urgent situations.

## Introduction

Approximately 644,000 individuals, constituting 0.7% of the population aged 12-35 in the United States, are expected to have some type of coronary artery anomaly (CAA) [1,2]. In another study, CAAs were found in 1,686 patients (1.3% incidence) undergoing coronary arteriography at the Cleveland Clinic Foundation from 1960 to 1988 [3]. A meta-analysis by D’Ascenzi et al. reported that the anomalous origin of coronary arteries accounts for 7.2% of sudden cardiac death (SCD) among athletes and 1.9% of SCD among non-athletes [4].

Anomalous right coronary artery (ARCA) can originate from a variety of sites, such as the descending thoracic aorta, left main coronary, left circumflex, above/from the left sinus of Valsalva, the pulmonary arteries, or even below the aortic valve [3,5]. Age at presentation is quite variable in patients with ARCA, as often they go undiagnosed [6]. Certain morphological and pathophysiological factors can increase the likelihood of SCD in anomalous coronary arteries [7]. Such morphological factors encompass characteristics such as a slit-like ostium, acute-angle takeoff, and a lengthy intramural course, while pathophysiological factors involve vasospasm, accelerated atherosclerosis, and abnormal coronary flow reserve due to endothelial dysfunction [8,9]. Together, these elements may contribute to myocardial ischemia and SCD. Nonetheless, exercise remains a persistent physiological occurrence associated with SCD in ARCA [10].

## Case presentation

A 22-year-old African American man with no significant past medical history collapsed suddenly at his workplace. He was working at a grocery store and, reportedly, was not in the middle of strenuous activity at the time of the collapse. The patient was found pulseless, and cardiopulmonary resuscitative measures were initiated immediately by bystanders, and 911 was called. Upon arrival at emergency medical services (EMS), the patient was found to have ventricular fibrillation. He was electrically cardioverted three times on the site by EMS before regaining a regular sinus rhythm.

At the time of presentation to our hospital, the patient was intubated. His family arrived with the patient and reported a childhood history of asthma only, and no other diagnosed illnesses were reported. The family also denied any recent illness. They reported the patient to be a perfectly healthy young man who does not smoke, drink alcohol, or use illicit drugs. No family history of heart disease was identified on the paternal side; however, the maternal side of the family history was unknown.

**Figure 1:**
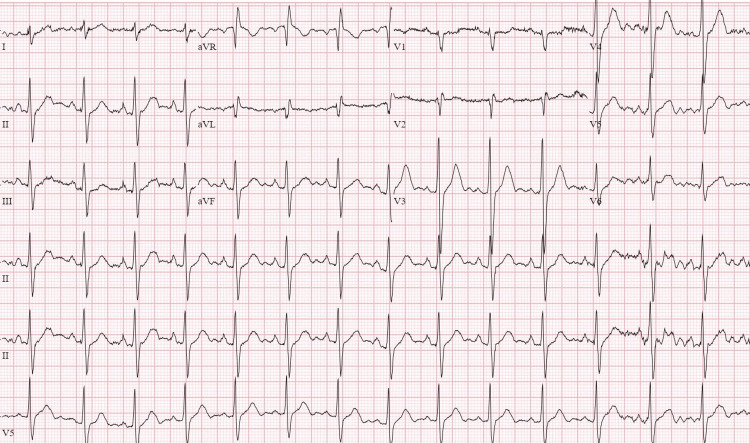
EKG on the day of presentation shows a normal sinus rhythm without significant ST changes EKG: electrocardiogram

Laboratory workup at the time of admission showed mild hypokalemia of 3.3 mmol/L (N: 3.5-5.1), lactate of 6.9 mmol/L (N: 0.4-2.0), blood glucose of 223 mg/dL (N: 74-106), and mildly elevated creatinine at 1.2 mg/dL (N: 0.55-1.02) with a normal estimated glomerular filtration rate. Troponins were within the normal range on the day of presentation. The urine drug screen was negative. No significant abnormality was identified in the rest of the admission labs. The EKG from the day of admission is shown below (Figure 1).

An echocardiogram obtained on admission showed normal left ventricular systolic function with an ejection fraction of 55%. No regional wall motion abnormality was identified. Right ventricular systolic function was mildly reduced. The right and left atria were of normal size. No pericardial effusion or valvular abnormality was noted.

**Figure 2:**
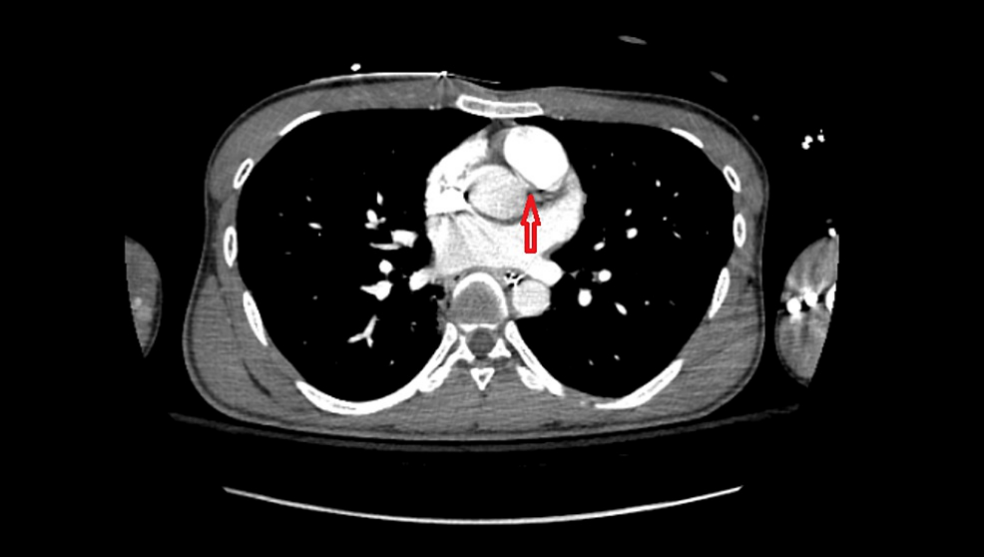
CT scan of the chest; note the vascular structure (arrow pointing) coursing between the aorta and the pulmonary trunk CT: computed tomography

A CT scan was negative for any significant airway or lung parenchymal pathology; however, when focused on the cardiac images, a possible inter-arterial vascular structure could be visualized coursing between the aorta and the pulmonary truck. This raised suspicion of the presence of an anomalous coronary artery in the patient. A labeled image of the CT scan with an arrow pointing at the said artery is shown below (Figure 2).

**Figure 3:**
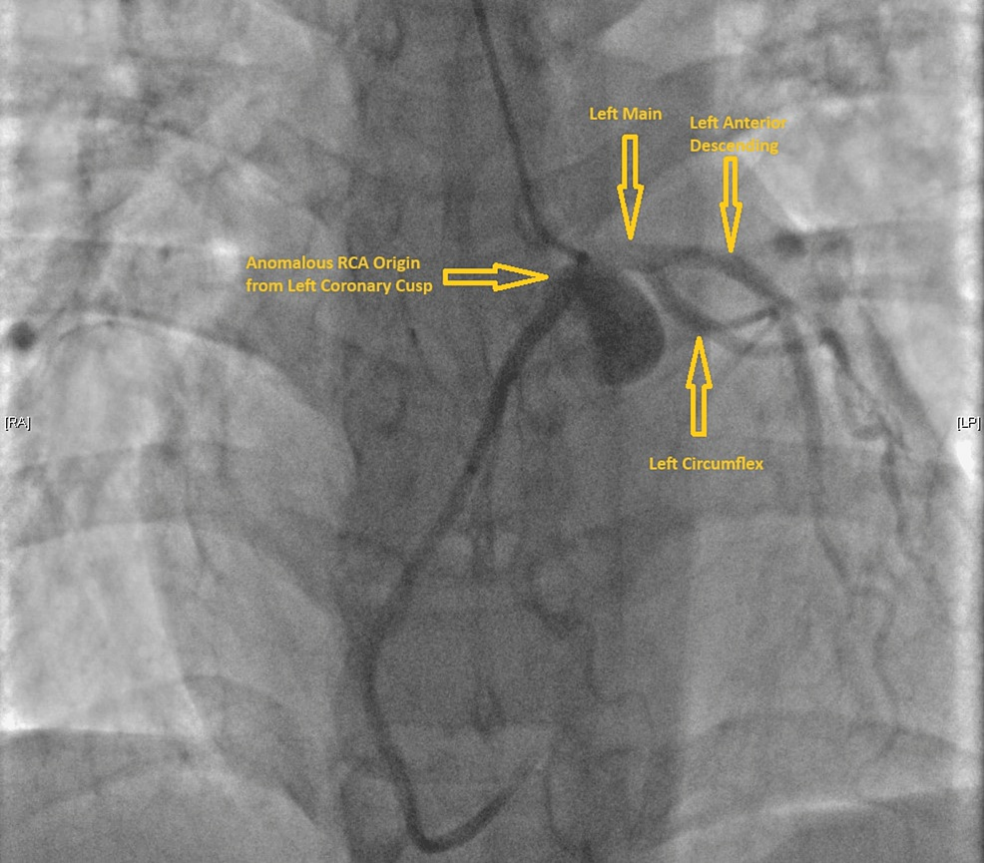
Cardiac catheterization image showing an anomalous RCA originating from the left coronary cusp (labeled) RCA: right coronary artery

Our cardiology department was involved in the care of the patient. After evaluation by cardiology, a decision was made to perform left-heart catheterization to investigate the etiology of sudden cardiac arrest. A left cardiac catheterization was performed on day 3 of hospitalization. Results of left heart catheterization (LHC) did not reveal any significant obstructive disease involving coronary arteries; however, an anomalous takeoff of the right coronary artery (RCA) from the common ostium of the left coronary cusp was confirmed on LHC. An image obtained during the cardiac catheterization showing the abnormal takeoff is provided below (Figure 3) for reference.

Even though cardiac catheterization confirmed the anomalous origin of the RCA, a cardiac MRI was recommended next by cardiology to highlight the route of the abnormally originating coronary artery. Cardiac MRI could not be performed since this imaging modality is unavailable at our facility. Therefore, a cardiac CT scan was performed next. A cardiac CT scan showed an anomalous RCA coursing between the aorta and pulmonary trunk. Due to technical difficulties at the time of publication, we are unable to include images of cardiac CT in our report.

We had thorough discussions with the patient and the family, explaining the nature of the congenital abnormality and the available treatment options. Although various surgical options are available to correct anomalous coronary artery courses, the surgical expertise at our facility is best familiar with coronary artery bypass grafting. After obtaining informed consent from the patient and family, a decision was made to proceed with coronary artery bypass grafting. The patient underwent coronary artery bypass grafting utilizing the saphenous vein graft. The patient recovered well post-surgery, and there were no postoperative complications reported. After discharge from the hospital, the patient followed up with an outpatient cardiology clinic. At a two-year outpatient follow-up, the patient continued to do well and remained symptom-free.

## Discussion

CAAs are a potentially life-threatening congenital disease that, while rare, is likely underrepresented due to the large, mostly asymptomatic population. It is even more uncommon to see SCD as the presenting symptom of these illnesses. An ARCA is usually diagnosed incidentally during angiography or at autopsy [11]. ARCA with an inter-arterial course (ARCA-IA) predominantly affects young male adults and has a higher predisposition to SCD, especially during exercise. The onset of symptoms probably occurs when the ischemic threshold has been surpassed. This likely occurs when the increasing size of either the aorta or pulmonary artery combines with an increased adrenergic surge to produce mechanical compression of the anomalous coronary artery [3]. Diagnosis is often made with multidetector CT or coronary MRI in combination with conventional angiography for precise anatomical information [1,12].

Early and accurate diagnosis of an anomalous coronary artery is imperative for timely intervention, when necessary if discovered early. Unfortunately, malignant coronary artery diseases often present with acute coronary syndromes, myocardial infarction, or SCD, which can make early diagnosis difficult. However, if discovered early, treatment options are available. Current literature provides examples of patients who have presented with a wide spectrum of symptoms, many of which are SCDs. Many of these cases are preceded by exertion causing an adrenergic surge, which is not seen in our case. This implies a certain population of patients with ARCA-IA may develop spontaneous, sudden cardiac arrest without provocation.

Available treatment options, all of which are viable in different clinical scenarios, include surgical revascularization, which is the recommended treatment option for all symptomatic patients [1]. These available surgical treatment options include surgical unroofing (the most common treatment option employed), coronary artery reimplantation and translocation, and coronary artery bypass grafting (with or without ligating the anomalous artery) [13,14]. PCI with stenting is an available treatment option for patients who are poor surgical candidates and usually provides symptomatic relief [12]. Further research should be undertaken to determine the long-term survival of patients who undergo surgical intervention versus patients who undergo PCI with stenting. Certain retrospective studies have found that patients who have been found to have an anomalous origin of the coronary arteries that arise from the opposite sinus of Valsalva have similar outcomes when surgical management is compared to medical management alone [14].

## Conclusions

In our case, rapid diagnosis and treatment were provided, despite the patient’s initial presentation of sudden cardiac arrest. Coronary artery bypass grafting was provided for this patient, as this was the most appropriate treatment option available at his treatment facility based on current literature. Additionally, the patient has had an excellent clinical outcome one year postoperatively, with no further symptoms since treatment. The patient will continue to be monitored clinically by his cardiologist, and time will tell if symptoms eventually recur. However, his clinical success to date supports surgical intervention as a viable option for patients with ARCA-IA, especially in an emergency setting. Further research can elucidate if this treatment option can be extrapolated to a larger population of all patients with an anomalous origin of a coronary artery.
